# Neuropsychiatric manifestations among HIV-1 infected African patients receiving efavirenz-based cART with or without tuberculosis treatment containing rifampicin

**DOI:** 10.1007/s00228-018-2499-0

**Published:** 2018-07-12

**Authors:** Sabina Mugusi, Eliford Ngaimisi, Mohammed Janabi, Ferdinand Mugusi, Omary Minzi, Eric Aris, Muhammad Bakari, Leif Bertilsson, Juergen Burhenne, Eric Sandstrom, Eleni Aklillu

**Affiliations:** 1 Department of Clinical Pharmacology, School of Medicine, Muhimbili University of Health and Allied Sciences (MUHAS), Dar es Salaam, Tanzania; 2 Department of Clinical Pharmacy and Pharmacology, School of Pharmacy, Muhimbili University of Health and Allied Sciences, Dar es Salaam, Tanzania; 3 Department of Internal Medicine, Muhimbili National Hospital, Dar es Salaam, Tanzania; 4 Department of Internal Medicine, School of Medicine, Muhimbili University of Health and Allied Sciences (MUHAS), Dar es Salaam, Tanzania; 5 Division of Clinical Pharmacology, Department of Laboratory Medicine, Karolinska University Hospital-Huddinge, Karolinska Institute, Stockholm, Sweden; 6 Department of Clinical Pharmacology and Pharmaco-epidemiology, University of Heidelberg, Heidelberg, Germany; 7 Department of Clinical Science and Education, Infectious Disease Unit, Karolinska Institutet, Södersjukhuset, Stockholm, Sweden

**Keywords:** Neuropsychiatric manifestations, HIV, Tuberculosis, Efavirenz, Rifampicin, *CYP2B6*

## Abstract

**Purpose:**

Efavirenz-based combination antiretroviral therapy (cART) is associated with neuropsychiatric adverse events. We investigated the time to onset, duration, clinical implications, impact of pharmacogenetic variations, and anti-tuberculosis co-treatment on efavirenz-associated neuropsychiatric manifestations.

**Methods:**

Prospective cohort study of cART naïve HIV patients with or without tuberculosis (HIV-TB) co-infection treated with efavirenz-based cART. Rifampicin-based anti-tuberculosis therapy was initiated 4 weeks prior to efavirenz-based cART in HIV-TB patients. Data on demographic, clinical, laboratory, and a 29-item questionnaire on neuropsychiatric manifestations were collected for 16 weeks after cART initiation. Genotyping for *CYP2B6*, *CYP3A5*, *SLCO1B1*, and *ABCB1* and quantification of efavirenz plasma concentration were done on the 4th and 16th week.

**Results:**

Data from 458 patients (243 HIV-only and 215 HIV-TB) were analyzed. Overall incidence of neuropsychiatric manifestations was 57.6% being higher in HIV-only (66.7%) compared to HIV-TB patients (47.4%) (*p* < 0.01). HIV-only patients were more symptomatic, with proportionately higher grades of manifestations compared to HIV-TB patients. Median time to manifestations was 1 week after cART initiation in HIV-only and 6 weeks after anti-TB (i.e., 2 weeks after cART initiation) in HIV-TB patients. HIV-only patients had significantly higher efavirenz plasma concentrations at 4 weeks after cART compared to HIV-TB patients. No association of sex or genotype was seen in relation to neuropsychiatric manifestations. Risk for neuropsychiatric manifestations was three times more in HIV-only patients compared to HIV-TB (*p* < 0.01).

**Conclusions:**

Incidence of neuropsychiatric manifestations during early initiation of efavirenz-based cART is high in Tanzanian HIV patients. Risk of neuropsychiatric manifestations is lower in HIV patients co-treated with rifampicin containing anti-TB compared to those treated with efavirenz-based cART only.

**Electronic supplementary material:**

The online version of this article (10.1007/s00228-018-2499-0) contains supplementary material, which is available to authorized users.

## Introduction

Sub-Saharan Africa carries the overwhelming burden of human immunodeficiency virus (HIV) and acquired immuno-deficiency syndrome (AIDS). Tanzania, one of the sub-Saharan countries, is greatly affected by this epidemic with a prevalence of 5% among adults aged 15–64 years, with a higher prevalence among females (6.5 vs 3.5%) [1]. The estimated HIV and AIDS population in Tanzania in 2016–2017 was around 1.4 million patients. Tuberculosis (TB) is the leading cause of death among AIDS patients accounting for 30% of overall mortality in these patients. During 1983–2016, patients with TB have increased sixfold [2]. With nearly 50% of TB patients being co-infected with HIV, the use of anti-TB treatment and combination anti-retroviral therapy (cART) is inevitable, running a risk for drug-drug interactions. Clinicians fear potential drug interactions and worry about poor efficacy of the drugs when co-administered resulting in poor treatment outcomes of both HIV and TB [2, 3].

Since the introduction of efavirenz in 1999, it has become the cornerstone in cART at a fixed daily dose of 600 mg [3, 4]. Efavirenz, a potent non-nucleoside reverse transcriptase inhibitor (NNRTI), is the backbone of first-line antiretroviral regimen of cART in most resource-limited countries including Tanzania. Efavirenz use has been associated with development of neuropsychiatric adverse events with some studies reporting incidences as high as 60–90% in Caucasian and black African patients [5–10]. Efavirenz easily crosses the blood–brain barrier, making it a neuroactive drug thus implicating it in causing neuropsychiatric manifestations [11]. High efavirenz concentrations in cerebrospinal fluid may cause behavioral and psychiatric manifestations; hence, maintenance of an optimal plasma concentration is required to ensure a balance between neuropsychiatric toxicity and possible treatment failure [12, 13]. The most common efavirenz-associated neuropsychological manifestations include dizziness, headache, confusion, impaired concentration, agitation, amnesia, depersonalization, hallucinations, abnormal dreams, and insomnia [5, 14–16]. Symptoms tend to occur within the first several days of treatment and generally resolve within the first 4 weeks of therapy [10, 14]. The adverse events of efavirenz have been found to correlate with plasma levels where the recommended therapeutic range is 1000–4000 ng/mL. Levels higher than 4000 ng/mL are associated with increased efavirenz toxicity [17–19].

Efavirenz is metabolized in the liver mainly by *CYP2B6* enzyme and to a lesser extent by *CYP3A4/5* [20, 21]. Rifampicin, a potent inducer of these enzymes, reduces the plasma efavirenz concentrations [22]. P-glycoprotein and *OATP1B1* coded by *ABCB1* and *SLCO1B1* gene respectively play a key role in the transportation of anti-TB drugs including rifampicin. These genes are inducible by rifampicin and are polymorphic displaying wide inter-individual and inter-ethnic variation in enzyme or transporter activity. CYP2B6 516G > T (CYP2B6*6), a defective variant allele associated with high efavirenz plasma concentration, liver enzyme abnormality, central nervous system (CNS) adverse events, and altered enzyme inducibility, occurs at a higher frequency (up to 40%) in sub-Saharan African populations compared to other populations [9, 23–26] Thus, identifying the incidence and risk factors of efavirenz-associated neuropsychiatric manifestation is imperative in Africa, where HIV remains a major public health problem. Efavirenz-associated neuropsychiatric manifestation and the associated predisposing risk factors are not well characterized in sub Saharan Africans. In the present study, we conducted a prospective study to investigate the incidence and risk factors, time to onset, duration and clinical implications, impact of pharmacogenetic variations, and anti-tuberculosis co-treatment, on efavirenz-associated neuropsychiatric manifestations in HIV-infected patients with or without tuberculosis coinfection.

## Materials and methods

### Study population and setting

This was part of a larger prospective cohort study entitled “Optimization of HIV and TB co-treatment based on pharmacokinetic and pharmacogenetic aspects of drug-drug interactions between Rifampicin and Efavirenz”. The study was conducted between September 2007 and June 2010 at Muhimbili National Hospital (MNH), Infectious Disease Centre (IDC) and Mwananyamala Municipal Hospital all within Dar es Salaam city, Tanzania. Ethical approval for the study was obtained from the Institutional Review Board (IRB) of Muhimbili University of Health and Allied Sciences (MUHAS) in Dar es Salaam, Tanzania and by Karolinska Institutet in Stockholm, Sweden. Prior written informed consent was obtained from all study participants. Patients were recruited depending upon the disease conditions and type of treatment they received according to the national HIV and TB treatment guidelines during the study period. The cohort included patients with diagnosed HIV infection without TB co-infection, referred to as HIV-only as well as HIV patients co-infected with TB referred to as HIV-TB. The eligibility criteria were consented adults **≥** 18 years of age, with documented HIV infection who was naïve to cART, with a CD4 count **≤** 200 cells/μL. Pregnant women, prisoners, and patients with low Hb (≤ 8 g/dL) were excluded. Patients were followed up for 48 weeks for HIV-only and 52 weeks for HIV-TB at regular intervals. All patients received the usual routine care for HIV and TB.

HIV-only patients were initiated on an efavirenz-based cART regimen with two NRTI’s. In the HIV-TB patients, a rifampicin-based anti-TB therapy was initiated 4 weeks prior to the initiation of efavirenz-based cART. A verbal autopsy questionnaire was administered by the clinician to the relatives of the deceased for reported deaths during the study period to ascertain cause of death [27, 28]. Where available, information from the deceased’s death certificate was used to complete the verbal autopsy questionnaire.

### Data collection and laboratory analysis

Socio-demographic characteristics, history of present and past illnesses, and findings from a general physical examination were recorded using a case record form prepared for the study. Clinical and laboratory data was collected at baseline, and at other pre-determined intervals at weeks 1, 2, 4, 8, 12, 16, 24, 36, and 48 after cART initiation. The study physicians did clinical evaluations for any adverse events and patients’ progress at every clinical visit.

### Assessment of neuropsychiatric manifestations in HIV infected patients with or without tuberculosis co-infection

A formulated questionnaire was used to collect data on patients’ neuropsychiatric status at baseline (before cART initiation) and at weeks 1, 2, 4, 8, 12, and 16 after cART initiation. This 29-item questionnaire was formulated from different tools used by other research groups [29–31]. The questionnaire was reviewed by a psychiatrist and a neurologist who believed that it captured all symptomatology pertaining to efavirenz-related neuropsychiatric manifestations. The same questionnaire was used at all the study time points (0, 1, 2, 4, 8, 12, and 16 weeks) to be able to follow patients’ symptomatology over time. During the same visit, the clinicians examined patients for clinical signs of neuropsychiatric and other adverse events. Patients were counseled on the occurrence of the known potential neuropsychiatric manifestations of efavirenz and were reassured by the clinician and nurses that this would be transient; however, they were to report any such side effects to the clinician or nurse. Social support (treatment supporter) was highly recommended among all patients as a way to enhance adherence to cART.

### CYP2B6, CYP3A5, ABCB1, and SLCO1B1 genotyping

Genomic DNA was isolated from peripheral blood leukocytes using QIAamp DNA Maxi Kit (QIAGEN GmbH. Hilden. Germany). Genotyping for SNPs were done by real**-**time PCR using pre-developed Taqman assay reagents for allelic discrimination (Applied Biosystems Genotyping Assays). Genotyping for SLCO1B1 388A > G (rs2306283) and 521 T > C (rs4149056) was done using LightCycler®**-**based method as described previously [32]. Haplotype analysis was done using Haploview v.4.1 software.

### Quantification of plasma efavirenz concentration

On weeks 4 and 16 of efavirenz-based cART, blood was collected at 16-h post efavirenz dosing for the determination of efavirenz and its metabolite concentration. Determination of plasma efavirenz and 8-hydroxyefavirenz concentrations by LC/MS/MS was performed as described previously [26, 33].

### Case definition

At baseline neuropsychiatric symptoms and a medical history of any psychiatric disorders were assessed. For the purpose of our study, identification of neuropsychiatric manifestations of efavirenz was based on the presence of any form of manifestation during the 16-week follow-up after cART was initiated. Severity of neuropsychiatric manifestations was also recorded based on the WHO-DAIDS grading system for monitoring toxicities adopted in the National HIV treatment guidelines; Mild symptoms (grade I–alteration causing no or minimal interference with usual social and functional activities), moderate symptoms (grade II–alteration causing greater than minimal interference with usual social and functional activities), severe symptoms (grade III–Alteration causing inability to perform social and functional activities), and very severe/potentially life threatening (grade IV–behavior potentially harmful to self or others, e.g., suicidal or homicidal ideation or attempt, psychosis, or causing inability to perform basic self-care functions).

### Statistical methods

Clinical assessment, neuropsychiatric assessment, and laboratory results recorded into a Microsoft Access database were analyzed using Statistical Package for the Social Sciences (PASW–former SPSS) version-18 and R version 2.9.2 (R Foundation for Statistical Computing, Vienna, Austria). Based on differences in analysis of neuropsychiatric manifestations in previous published research due to lack of a gold standard, we grouped the 29-item symptoms into three groups namely group 1 (anxiety-depressive symptoms)—which contained symptoms of anxiety, depression, stress, and problems with daily concentration; group 2 (sleep disturbances) contained symptoms of abnormal dreams and sleep disturbances; and group 3 (dizziness and psychotic symptoms) had symptoms of dizziness and confusion. The incidence, time to onset, duration, clinical implications, and impact of pharmacogenetics on neuropsychiatric manifestations of efavirenz among HIV-only and HIV-TB coinfected patients were assessed using descriptive statistics. Baseline demographic, clinical characteristics, and laboratory values were tested with the independent *t* test and χ^2^-test. Univariable followed by multivariable Cox proportional hazards regressions were performed to determine the risk factors for neuropsychiatric manifestations of efavirenz. The variables included in the multivariable model were those with either a theoretical importance or ones with a *p* value < 0.05 in the univariable models. *p* value of < 0.05 was considered statistically significant.

## Results

### Neuropsychiatric manifestations

A total of 486 patients (255 HIV-only and 231 HIV-TB) were recruited into the cohort. Data from 458 patients (243 HIV-only and 215 HIV-TB) with documentation on the neuropsychiatric manifestations were used for analysis. Socio-demographic, clinical, and laboratory characteristics of patients with and without neuropsychiatric manifestations after initiation of efavirenz-based cART is presented in Table 1. Of the 458 patients, 264 (57.6%) had some form of neuropsychiatric manifestations during the 16-week study period. Significant differences in the incidence of neuropsychiatric manifestations were seen between HIV-only compared to the HIV-TB patients being higher in HIV-only patients treated with efavirenz-based cART compared to those co-treated with rifampicin-based anti-TB (66.7 and 47.4% respectively) (*p* < 0.01). Kaplan-Meier plot indicating cumulative hazard for the development of neuropsychiatric manifestations stratified by disease status is presented in supplementary materials (Supplementary Fig. 1). Significant differences were also noted among the different WHO clinical stages (more patients with neuropsychiatric manifestations being in WHO stages III and IV) and the NRTI combination of cART used among these patients (more patients with neuropsychiatric manifestations having a zidovudine-based NRTI, *p* < 0.01). Smoking (*p* = 0.03) and comorbidities other than TB (*p* < 0.01) were also significant. These comorbidities included oral candidiasis, Kaposis Sarcoma, pruritic papular eruptions (PPE), herpes simplex infections, and diabetes mellitus (Table 1).

**Table 1:** Socio-demographic, baseline clinical, and laboratory characteristics of patients with and without neuropsychiatric manifestations after initiation of efavirenz-based cART

Characteristic		No neuropsychiatric manifestations (*n* = 194)	Yes neuropsychiatric manifestations (*n* = 264)	*p* value
Group	HIV only	81 (33.4%)	162 (66.6%)	< 0.01
	TB-HIV	113 (52.6%)	102 (47.4%)	
Sex	Females	112 (42.9%)	149 (57.1%)	0.78
	Males	82 (41.6%)	115 (58.4%)	
Age [Mean ± SD] (years)		39.48 (9.67)	40.28 (9.17)	0.37
BMI [Mean ± SD] (kg/m^2^)		21.10 (4.32)	20.99 (4.11)	0.78
Marital status	Single, divorced or widowed	100 (40.7%)	146 (59.3%)	0.43
	Married or cohabiting	94 (44.3%)	118 (55.6%)	
Education status	Illiterate, able to read or write or primary	150 (42.6%)	202 (57.4%)	0.84
	Secondary or tertiary	44 (41.5%)	62 (58.5%)	
Karnofsky scores	90–100%	142 (41.4%)	201 (58.6%)	0.47
	≤ 80%	52 (45.2%)	63 (54.8%)	
WHO clinical stages	Stage II	72 (36.9%)	123 (63.1%)	< 0.01
	Stage III	117 (48.9%)	122 (51.1%)	
	Stage IV	5 (20.8%)	19 (79.2%)	
cART initiated	d4T + 3TC + EFV	76 (51.7%)	71 (48.3%)	< 0.01
	AZT + 3TC + EFV	114 (37.4%)	191 (62.6%)	
Alcohol consumption	Yes	44 (42.3%)	60 (57.7%)	0.99
	No	150 (42.4%)	204 (57.6%)	
Smoking	Never smoked	132 (39.3%)	204 (60.7%)	0.03
	Current/ex-smoker	62 (50.8%)	60 (49.2%)	
Comorbidities	No	182 (44.5%)	227 (55.5%)	< 0.01
	Yes	12 (24.5%)	37 (75.5%)	
Laboratory parameters				
Median baseline CD4 Cell counts [IQR] (cells/μL)		87.5 [124]	95 [114]	0.95
Mean baseline viral load, log 10 [± SD] (copies/mL)		5.2 [0.94]	5.1[0.85]	0.69
Baseline neuropsychiatric manifestations				
Any neuropsychiatric manifestation at baseline	HIV only	217 (89.6%)	26 (10.6%)	0.11
	HIV-TB	201 (93.3%)	14 (6.7%)	

*cART* combination antiretroviral therapy, *BMI* body mass index, *d4T* stavudine, *3TC* lamivudine, *EFV* efavirenz, *AZT* zidovudine, *IQR* interquartile range, *SD* standard deviation

Based on the different groups, the dominating symptoms in group 1 (anxiety-depressive symptoms) were feeling tired, poor appetite, and feeling down, and depressed. Symptoms in group 2 (sleep disturbances) of abnormal dreams and sleep disturbances were very common. Dominating symptoms in group 3 (dizziness and psychotic symptoms) were headache and symptoms relating to dizziness. Previously reported symptoms from literature report the association of efavirenz-based cART with CNS adverse events mainly abnormal dreams and sleep disturbances (group 2) were noted in this study. The incidence of any group 2 (sleep disturbances) adverse events which was significantly higher in HIV-only patients compared to HIV-TB patients co-treated with rifampicin (45 vs 25%), in particular, were vivid dreams (25 vs 13%), nightmares (28 vs 12%), sleep disturbances (25 vs 10%), and hallucination (5 vs 2%). The differences in symptoms and severity grades of neuropsychiatric manifestations between HIV-only and HIV-TB patients are presented in the supplementary materials (Supplementary Table 1).

The third (33.3%) of the HIV-only compared to over half (52.6%) of those with HIV-TB did not present with any neuropsychiatric manifestations. However, for symptomatic patients, significantly higher severity was reported in general in those with HIV-only compared to those with HIV-TB (*p* < 0.01). The median time to neuropsychiatric manifestations was 2 weeks. However, in the HIV-only patients, the median time was 1 week after cART initiation while in patients with HIV-TB this was seen at 6 weeks (i.e., 2 weeks after cART was initiated). This difference in time to manifestation is significant (*p* < 0.01). In HIV-only patients, peak manifestations started at week 1 (57%) after which these started declining to 54% at 2 weeks, 43% at 4 weeks, and 20% at 8 weeks. By weeks 12 and 16 after cART initiation in the HIV-only patients, the proportions of neuropsychiatric manifestations dropped to 7 and 5% respectively. In contrast, manifestations among HIV-TB were 2.6% in week 1 (on anti-TB therapy only), peaked between weeks 4 (at cART initiation), 6 (2 weeks after cART initiation), and 8 (4 weeks after cART initiation (38, 43 and 40%) respectively). At weeks 12 and 16 after cART initiation, the proportion of patients with neuropsychiatric manifestations was 7 and 4% respectively among the HIV-TB patients.

### Neuropsychiatric manifestations and efavirenz concentrations

Plasma efavirenz concentrations were determined from 306 and 236 study participants at weeks 4 and 16 respectively. No significant differences were seen in median efavirenz concentrations among patients with and without neuropsychiatric manifestations at 4 weeks (1779 ng/mL interquartile range {IQR} = 3041 ng/mL and 1733 ng/mL {IQR = 2114 ng/mL} respectively) and at 16 weeks (1590 ng/mL {IQR = 1740 ng/mL} and 1422 ng/mL {IQR = 1599 ng/mL}) respectively. Similarly, no significant differences were seen in the efavirenz metabolites (8-hydroxyefavirenz) among patients with and without neuropsychiatric manifestations at either weeks 4 or 16 after cART initiation.

**Fig. 1:**
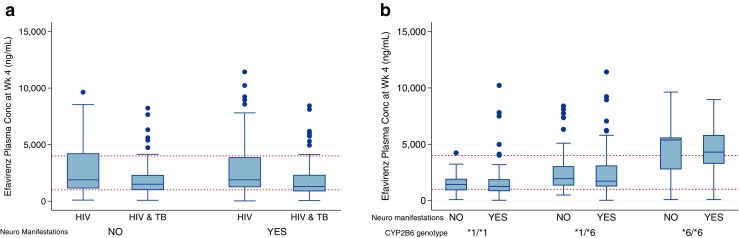
**a** Box plots showing efavirenz plasma concentrations at 4 weeks after cART initiation measured 16 h after the daily 600 mg efavirenz dosing among the HIV-only and the presenting with and without neuropsychiatric manifestations. **b** Box plots showing median efavirenz plasma concentrations at 4 weeks after cART initiation measured 16 h after the daily 600 mg efavirenz dosing among the patients with and without neuropsychiatric manifestations based on the *CYP2B6* genotype

When stratified by disease status (HIV-only and HIV-TB), there were statistical differences in the median efavirenz concentrations at 4 weeks in patients who presented with neuropsychiatric manifestations (median 1994 ng/mL {IQR = 3080 ng/mL} in HIV-only vs 1217 ng/mL {IQR = 1726 ng/mL} in HIV-TB patients) (*p* = 0.02) (Fig. 1a). No such difference was seen for efavirenz concentrations at 16 weeks after cART initiation. There were no significant sex differences among patients with and without neuropsychiatric manifestations; however, differences in sex was seen for efavirenz concentrations at weeks 4 and 16 among patients who presented with neuropsychiatric manifestations. Females with neuropsychiatric manifestations had significantly higher median plasma efavirenz concentrations compared to the males at 4 weeks (1914 ng/mL {IQR = 3151} vs 1464 ng/mL {IQR = 1752} (*p* = 0.01)) and at 16 weeks (1653 ng/mL {IQR = 2396} vs 1264 ng/mL {IQR = 1625} (*p* = 0.02)).

The direct correlation between the efavirenz concentrations and the severity of symptoms at 4 weeks after cART initiation was done. In HIV-only patients, the median efavirenz concentrations were non-significantly higher in those with none, mild, moderate, and severe symptoms (1892 {IQR = 2975}, 1311 {IQR = 907}, 2653 {IQR = 2286} and 2041 ng/mL {IQR = 3807}) compared to (1502 ng/mL {IQR = 1417}, 1252 ng/mL {IQR = 731}, 1057 ng/mL {IQR = 2248} and 1764.8 ng/mL {IQR = 2287}) among the HIV-TB respectively. The HIV-only patients had more symptoms at week 4 compared to the HIV-TB patients where 3.9% of the HIV-only patients had between 10 and 14 symptoms whereas none of the HIV-TB patients had over 10 symptoms out of the 29-symptom questionnaire.

### Neuropsychiatric manifestations and genotypes

A total of 341 samples (74.5%) were analyzed. For CYP2B6*6, 41.6% were *1/*1, 42.5% were *1/*6 and 15.8% were *6/*6. No significant differences in the genotype distribution among those with and without neuropsychiatric manifestations were seen. Likewise, no differences were noted in the genotype frequencies among the HIV-only patients compared to those with HIV-TB. The median efavirenz concentrations at 4 weeks for patients with neuropsychiatric manifestations were 1253 ng/mL {IQR = 1066}, 1721.7 ng/mL {IQR = 1887 ng/mL} and 4501 ng/mL {IQR = 2495 ng/mL} for CYP2B6*1/*1, *1/*6 and *6/*6 respectively. Similar differences were noted for the median efavirenz concentrations among patients without neuropsychiatric manifestations which were 1427.4 ng/mL {IQR = 1076}, 1945.5 ng/mL {IQR = 2009}, and 5381.1 ng/mL {IQR = 4647} for *1/*1, *1/*6, and *6/*6 respectively (Fig. 1b). Median efavirenz concentrations at 16 weeks after cART initiation among patients with and without neuropsychiatric manifestations presented a similar pattern among the different genotypes where patients with *1/*1 genotypes had significantly lower efavirenz concentrations compared to those with *6/*6 genotypes. Genotyping for CYP3A5*3, *6, and *7 were done, and subjects were classified based on number of *CYP3A5* functional allele (*1). For *CYP3A5,* 26.4% were carriers of no CYP3A5*1 allele, whereas 48.4 and 25.2% were carriers of one and two CYP3A5*1 alleles respectively. No significant differences between the different *CYP3A5* genotype groups were noted among those with and without neuropsychiatric manifestations. No associations were noted for *MDR1* and *SLCO1B1* in causing these manifestations.

### Predictors for neuropsychiatric manifestations

Univariable analysis showed that HIV-only, WHO grades III and IV, non-smokers, comorbidity other than TB, and NRTI combination used were predictors for developing neuropsychiatric manifestations. When analyzed by the three different groups, the prominent predictor for group 1 (anxiety-depressive symptoms) (hazards ratio {HR} 3.350; 95% confidence interval {95% CI} 1.732–6.479) and group 2 (sleep disturbances) (HR 4.553; 95% CI 2.007–10.32) neuropsychiatric manifestations were HIV-only patients on efavirenz-based cART without rifampicin. Predictors for group 3 manifestations (symptoms of dizziness and confusion) were like the other groups HIV-only disease (HR 5.171; 95% CI 2.328–11.484) and a stavudine-based NRTI regimen (HR 1.843; 95% CI 1.016–3.343).

Interestingly, plasma efavirenz concentrations at weeks 4 or 16 of cART and *CYP2B6* were not found to be predictors of neuropsychiatric manifestations in our study. Variables with *p* < 0.05 in the univariable analysis and those that were deemed as theoretical predictors for neuropsychiatric manifestations were included into the multivariable model (Table 2). Multivariable analysis was done for all-cause neuropsychiatric manifestation, which showed that the predictor for neuropsychiatric manifestation (regardless of the group) was HIV-only patients on efavirenz-based cART without concomitant rifampicin (HR 2.82, 95% CI 1.493–5.324).

**Table 2:** Univariate and multivariate correlates for neuropsychiatric manifestations of efavirenz-based cART among HIV patients

	Univariate analysis		Multivariate analysis	
	HR (95% CI)	*p* value	HR (95% CI)	*p* value
Treatment group (ref – HIV-TB patients)	2.05 (1.593–2.628)	< 0.01	2.83 (1.498–5.337)	< 0.01
Sex (ref – males)	1.01 (0.793–1.291)	0.92	0.77 (0.524–1.117)	0.16
Age groups (ref – > 35)	0.85 (0.658–1.094)	0.21		
Age	1.01 (0.992–1.017)	0.46		
Marital status (ref – married)	1.07 (0.837–1.360)	0.59		
BMI	0.99 (0.968–1.028)	0.89		
BMI groups (ref – > 25.1)		0.19		0.29
< 18.1	0.88 (0.614–1.259)	0.48	1.28 (0.801–2.049)	0.30
18.1–25	0.74 (0.524–1.053)	0.09	0.94 (0.612–1.442)	0.77
Smoking (ref – yes smoking)	1.37 (1.026–1.826)	0.03	1.29 (0.801–2.072)	0.29
Alcohol (ref – no alcohol use)	1.02 (0.768–1.366)	0.87		
Comorbidity (ref – no)	1.81 (1.277–2.567)	< 0.01	0.72 (0.395–1.309)	0.28
Karnofsky scores (ref – < 80%)	1.09 (0.820–1.445)	0.56		
cART (ref – d4T)	1.55 (1.177–2.032)	< 0.01	1.38 (0.871–2.180)	0.17
WHO stages (ref – stage IV)		< 0.01		0.86
Stage II	0.78 (0.480–1.264)	0.31	0.80 (0.319–2.031)	0.64
Stage III	0.47 (0.288–0.759)	< 0.01	0.90 (0.348–2.333)	0.83
Baseline CD4 groups (ref – > 101 cells/μL)	1.01 (0.793–1.286)	0.94		
CD4 (cells/μL)	1.00 (0.998–1.001)	0.66	1.00 (0.999–1.004)	0.17
Baseline viral load log	1.00 (0.936–1.072)	0.95		
Log EFV conc at week 4	0.98 (0.852–1.130)	0.79	0.83 (0.582–1.177)	0.29
EFV at week 4 (ref – > 4000 ng/mL)		0.76		
< 1000 ng/ml	1.04 (0.619–1.727)	0.87		
1000–4000 ng/mL	0.92 (0.645–1.308)	0.64		
Log EFV conc at week 16	1.02 (0.852–1.130)	0.79		
EFV at week 16 (ref – > 4000 ng/mL)		0.85		
< 1000 ng/ml	1.03 (0.619–1.727)	0.89		
1000–4000 ng/mL	1.12 (0.718–1.741)	0.62		
*CYP 2B6* (ref – *1/*1)		0.99		0.82
*1/*6	0.99 (0.730–1.332)	0.93	0.93 (0.572–1.502)	0.76
*6/*6	1.05 (0.705–1.561)	0.81	0.87 (0.542–1.381)	0.54
*CYP3A5* (ref – *0/*0)		0.78		
*0/*1	1.11 (0.794–1.551)	0.54		
*1/*1	1.13 (0.776–1.657)	0.51		
Log10 8-OH EFV week 4	1.04 (0.870–1.240)	0.68		
Log10 MR EFV week 4	0.97 (0.848–1.028)	0.89		
Log10 8-OH EFV week 16	1.02 (0.866–1.196)	0.83		
Log10 MR EFV week 16	1.12 (0.942–1.324)	0.20		

*cART* combination antiretroviral therapy, *Ref* reference variable, *BMI* body mass index, *EFV* efavirenz, *d4T* stavudine, *VL* viral load, *8-OH* 8 hydroxyefavirenz, *MR* efavirenz/8-OH efavirenz ratio, HR hazard ratio

### Neuropsychiatric manifestations and treatment outcome

A total of five HIV-only patients (1.1%) had their efavirenz discontinued due to severe neuropsychiatric manifestations and refusal to continue taking efavirenz. Of these five patients, we had DNA samples from three patients for genotyping. All three patients were heterozygous CYP2B6 *1/*6, and for the *CYP3A5*, one patient had no functional CYP3A5*1, and two patients had only one CYP3A5*1 allele. Samples available from two of the three patients for plasma efavirenz concentration at week 4 were 2981 and 3153 ng/mL respectively.

Mortality analysis showed 45 of 458 patients (9.8%) died of whom 29 patients (6.3%) had neuropsychiatric manifestations. Causes of death were not related to the neuropsychiatric manifestations. There was a good, gradual and comparable immunological outcome of cART evidenced by an increase in the mean CD4 cell counts at 12 weeks after cART initiation between those with and without neuropsychiatric manifestations (108 vs 114cells/μL). Despite the presence of neuropsychiatric manifestations, patient’s self-reported adherence to cART in both the HIV-only using cART alone and HIV-TB patients using both anti-TB and cART was over 95%.

## Discussion

In the present study, we found the incidence of efavirenz-associated neuropsychiatric manifestations relatively high (57.6%), similar to that reported from other studies in Sub-Saharan Africa and elsewhere [9, 15, 34]. Compared to HIV-only patients receiving efavirenz-based cART, those receiving an additional rifampicin containing anti-TB treatment have significantly lower rates of neuropsychiatric manifestations. HIV-only patients were more symptomatic and had significantly higher plasma efavirenz concentrations at 4 weeks after cART initiation compared to those with HIV-TB co-treated with rifampicin. Our findings of higher neuropsychiatric manifestations in HIV-only patients could be mainly due to higher efavirenz plasma concentration in this group compared to HIV-TB co-infected patients receiving efavirenz-based cART together with rifampicin-based anti-TB therapy. The HIV-TB patients had been treated with rifampicin-based anti-TB therapy for 4 weeks prior to the initiation of efavirenz-based cART. This is in line with previous reports stating that rifampicin, a potent *CYP2B6* and *CYP3A5* inducer, lowers efavirenz plasma concentration particularly during early initiation of efavirenz-based cART but has no significant long-term effect [9, 35–37].

Peak timing for incidences of CNS symptoms concurs with efavirenz pharmacokinetic profile [9]. Stratified by treatment group (HIV-only and HIV-TB), the median efavirenz concentrations at 4 weeks were significantly higher in patients who presented with neuropsychiatric manifestations, but in a multivariable analysis considering all study participants, efavirenz plasma concentration was not a significant predictor for neuropsychiatric symptoms. Efavirenz displays a longer half-life, and its plasma concentration steadily increases soon after treatment initiation. Time-to-peak plasma concentrations occur approximately 3–5 h, and steady-state plasma concentrations reach in 6–10 days [5]. However, multiple doses of efavirenz for 10 days resulted in a lower than predicted extent of accumulation (22–42% lower) and a shorter terminal half-life of 40–55 h (single dose half-life 52–76 h) [5]. It is well known that efavirenz induces both *CYP2B6* and *CYP3A5* lowering its plasma concentration over time [26, 33]. Similarly, efavirenz-associated neuropsychiatric symptoms began early in therapy but resolve promptly by week 4 [38]. Accordingly, in our HIV-only patients, the median time to manifestations was 1 week after cART initiation while in patients with HIV-TB, this was seen at 6 weeks (i.e., 2 weeks after cART was initiated). Several studies reported significant association of high efavirenz plasma concentration and CYP2B6*6 genotype with neuropsychiatric manifestations [9, 18, 23, 38]. In our study, we measured efavirenz plasma concentration at 4 and 16 weeks after cART initiation but the peak CNS symptom occurred 1 or 2 weeks post efavirenz initiation in HIV-only and HIV-TB patients respectively. Such discrepancy between timing for efavirenz pharmacokinetic studies and peak CNS manifestation might be the reason for the observed lack of association between efavirenz plasma concentrations and the reported efavirenz associated sleep disturbances and hallucinations in our patients. Among the symptomatic patients, though not significant, a number of patients had low efavirenz concentrations. Alternatively, poor adherence to cART as seen by relatively low efavirenz metabolites (8-hydroxyefavirenz) despite high self-reported adherence among these patients might also be the reason.

Baseline age, BMI, and CD4 did not differ among patients with and without neuropsychiatric manifestations [23, 34]. Sex differences have been previously reported stating that women were more liable to have efavirenz-related toxicity compared to the men in the same ethnicity [39, 40]. Likewise, in our study, females had significantly higher plasma efavirenz concentrations compared to males though proportionately the number of neuropsychiatric manifestations between them was not significant. In symptomatic patients, those with CYP2B6 *6/*6 genotypes had significantly higher plasma efavirenz concentrations compared to those with *1/*6 and *1/*1. Wide between-patient variations in the mean efavirenz concentrations in our study could be explained in part by inter-individual variations in the *CYP2B6* activities. The risk of developing neuropsychiatric manifestations in HIV-only patients was three times more than that among HIV-TB patients using rifampicin-based anti-TB treatment with concomitant cART.

Group 1 symptoms of depression and anxiety seem to occur in both HIV-only and HIV-TB patients when they are on anti-TB alone therapy (before initiating efavirenz). These symptoms were mild, and not specific to known efavirenz neurotoxic side effects. These mild symptoms were responsive to formal and informal supportive counseling. The lower proportion of symptoms in HIV-TB patients could partially be explained by the frequent visits to the health center for their anti-TB therapy as part of Direct Observed Therapy short course (DOTS) allowing for more contact with the counselors [2]. The higher proportion of symptoms in the HIV-only could also be due to the stress brought on by psychological adjustment to recent HIV diagnosis.

HIV disease itself is a risk factor for neuropsychological impairments despite the HIV stage [41]. Patients with low CD4 nadir are also more likely to have these impairments as seen in our study where most of the patients had lower CD4 cell-counts with a larger proportion having counts below 100 cells/μL. Addition of efavirenz-based cART caused an increase in these symptoms in the first week of therapy. However, waning off of these symptoms in subsequent weeks could also be explained by the immune reconstitution as seen by increase in CD4 counts at 12 weeks, efavirenz auto-induction, and the use of concomitant rifampicin in the HIV-TB patients [25, 26].

Literature shows that neuropsychiatric symptoms occur early after initiating efavirenz (1 or 2 days after), and these symptoms spontaneously resolve in 4 weeks [16, 38, 42]. Our study shows that these manifestations occur within the first week of efavirenz initiation; however, we lacked the direct correlation of these manifestations with the plasma efavirenz concentrations measured at 4 or 16 weeks of cART and genotypes. Our results are similar to those seen in other studies that did not show a direct association [34, 43]; however, we measured the serum concentrations of efavirenz at the fourth week after cART. Other trials were able to make the association by measuring the efavirenz concentrations at the onset of the manifestations rather than later, where this association was no longer seen [9, 23, 38]. Limitation of our study includes that we assessed for any neuropsychiatric symptoms starting from baseline; however, we were unable to measure the concentrations until week 4. Following the WHO and the Tanzanian national HIV treatment guideline valid during the study period, only very sick patients with CD4 count < 200 cells were eligible for antiretroviral treatment. Thus, our study participants were treatment naïve very ill patients who were subjected to frequent blood sampling to monitor laboratory parameters including liver enzymes as part of the routine patient care measures [24]. Considering the patients clinical conditions and for ethical reason, we were not able to get blood sample for quantification of efavirenz plasma concertation until week 4 of ART, and some of the neuropsychiatric manifestations resolved during the first 4 weeks of ART. Hence, we cannot rule out the possibility of a correlation between neuropsychiatric manifestations and plasma efavirenz concentrations measured during the first 4 weeks of ART. However, as therapy continues, proportion of patients with neuropsychiatric manifestations decreased in both groups of patients. Despite this limitation, our exploratory study provides important information regarding the incidence and risk factors, time to onset, duration, and clinical implications, impact of pharmacogenetic variations, and anti-tuberculosis co-treatment, on efavirenz-associated neuropsychiatric manifestations in HIV-infected patients with or without tuberculosis coinfection.

## Conclusion

Despite a relatively high proportion of patients reporting neuropsychiatric manifestations attributed to efavirenz, these symptoms are transient and not severe. The use of rifampicin-containing anti-TB therapy concomitantly with efavirenz decreases the risk of developing neuropsychiatric manifestations up to three times compared to those on efavirenz-based cART. Plasma efavirenz concentrations at week 4 after cART initiation and the *CYP2B6* genotype are not positively associated with development of neuropsychiatric manifestations. Patients counseled on the neuropsychiatric side effects continue the drugs with positive anticipation of symptoms that resolve promptly. Social support may be helpful in alleviating most of the subjective neuropsychiatric manifestations.

## Electronic supplementary material

Supplementary Table 1 (DOCX 15 kb)

Supplementary Figure 1 (PNG 278 kb)

High resolution image (TIFF 94388 kb)
