# Supplementary material for: Neuropsychiatric manifestations among HIV-1 infected African patients receiving efavirenz-based cART with or without tuberculosis treatment containing rifampicin
Source: Eur J Clin Pharmacol. 2018 Jul 12;74(11):1405–15. doi: 10.1007/s00228-018-2499-0 (PMC6182598; doi:10.1007/s00228-018-2499-0)
Supplement: Supplementary file 1 — (DOCX 15 kb) [file 228_2018_2499_MOESM1_ESM.docx]

**Supplementary Table-1: Presentation of neuropsychiatric symptoms grade among HIV-only and HIV-TB co-infected patients on efavirenz based cART during the 16 weeks follow-up.**

| **Neuropsychiatric**  **Symptom** | **HIV only (n=243)** | | | | **HIV-TB (n=215)** | | | |
| --- | --- | --- | --- | --- | --- | --- | --- | --- |
|  | **None (%)** | **Mild (%)** | **Mod (%)** | **Sev (%)** | **None (%)** | **Mild (%)** | **Mod (%)** | **Sev (%)** |
| **Group 1: Anxiety, Depression, Stress and problems with Daily Concentration** | | | | | | | | |
| Feeling Tired | 124 (51.0) | 32 (13.2) | 79 (32.5) | 8 (3.3) | 158 (73.5) | 32 (14.9) | 22 (10.2) | 3 (1.4) |
| Poor Appetite | 133 (54.7) | 28 (11.5) | 64 (26.3) | 18 (7.4) | 154 (71.6) | 36 (16.7) | 16 (7.4) | 9 (4.2) |
| Feeling down and depressed | 199 (81.9) | 8 (3.3) | 33 (13.6) | 3 (1.2) | 199 (92.6) | 11 (5.1) | 5 (2.3) | 0 |
| Little Interest or Pleasure | 221 (90.9) | 11 (4.5) | 11 (4.5) | 0 | 201 (93.5) | 9 (4.2) | 5 (2.3) | 0 |
| Trouble concentrating | 225 (92.6) | 6 (2.5) | 12 (4.9) | 0 | 211 (98.1) | 2 (0.9) | 2 (0.9) | 0 |
| Feeling bad about self | 229 (94.2) | 8 (3.3) | 5 (2.1) | 1 (0.4) | 204 (94.9) | 9 (4.2) | 2 (0.9) | 0 |
| Anxious mood | 225 (92.6) | 7 (2.9) | 11 (4.5) | 0 | 212 (98.6) | 1 (0.5) | 2 (0.9) | 0 |
| Fears | 225(92.6) | 7 (2.9) | 11 (4.5) | 0 | 210 (97.7) | 3 (1.4) | 2 (0.9) | 0 |
| Tension | 232 (95.5) | 3 (1.2) | 8 (3.3) | 0 | 211 (98.1) | 3 (1.4) | 1 (0.5) | 0 |
| Moving or speaking slowly | 237 (97.5) | 3 (1.2) | 3 (1.2) | 0 | 210 (97.7) | 2 (0.9) | 3 (1.4) | 0 |
| Guilty feeling | 240 (98.8) | 3 (1.2) | 0 | 0 | 214 (99.5) | 1 (0.5) | 0 | 0 |
| Better off dead thoughts | 241 (99.2) | 2 (0.8) | 0 | 0 | 213 (99.1) | 1 (0.5) | 1 (0.5) | 0 |
| **Group 2: Abnormal dreams and sleep disturbances** | | | | | | | | |
| Nightmares | 176 (72.4) | 12 (4.9) | 52 (21.4) | 3 (1.2) | 188 (87.4) | 12 (5.6) | 14 (6.5) | 1 (0.5) |
| Vivid dreams | 182 (74.9) | 13 (5.3) | 44 (18.1) | 4 (1.6) | 188 (87.4) | 15 (7.0) | 11 (5.1) | 1 (0.5) |
| Sleep disturbances | 182 (74.9) | 21 (8.6) | 38 (15.6) | 2 (0.8) | 193(89.8%) | 9 (4.2) | 13 (6.0) | 0 |
| Trouble falling asleep at night | 184 (75.4) | 18 (7.4) | 40 (16.5) | 1 (0.4) | 195 (90.7) | 12 (5.6) | 8 (3.7) | 0 |
| Trouble staying awake | 194 (79.8) | 14 (5.8) | 35 (14.0) | 0 | 200 (93.0) | 10 (4.7) | 5 (2.3) | 0 |
| **Group 3: Dizziness and confusion** | | | | | | | | |
| Headache | 164 (67.5) | 39 (16.0) | 37 (15.2) | 3 (1.2) | 166 (77.2) | 38 (17.7) | 10 (4.7) | 1 (0.5) |
| Feeling light headed | 207 (85.2) | 16 (6.6) | 19 (7.8) | 1 (0.4) | 200 (93.0) | 14 (6.5) | 1 (0.5) | 0 |
| Room spinning feeling | 213 (87.7) | 22 (9.1) | 8 (3.3) | 0 | 208 (96.7) | 4 (1.9) | 3 (1.4) | 0 |
| Falling over feeling | 214 (88.1) | 19 (7.8) | 9 (3.7) | 1 (0.4) | 204(94.9) | 8 (3.7) | 3 (1.4) | 0 |
| Hallucination | 231 (95.1) | 3 (1.2) | 8 (3.3) | 1 (0.4) | 211 (98.1) | 1 (0.5) | 3 (1.4) | 0 |
| Elated mood | 241 (99.2) | 0 | 2 (0.8) | 0 | 214 (99.5) | 1 (0.5) | 0 | 0 |
| Unusual thought content | 241 (99.2) | 1 (0.4) | 1 (0.4) | 0 | 212 (98.6) | 3 (1.4) | 0 | 0 |
| Bizarre behavior | 241 (99.2) | 0 | 2 (0.8) | 0 | 213 (99.1) | 1 (0.5) | 1 (0.5) | 0 |
| Suspiciousness | 241 (99.2) | 0 | 2 (0.8) | 0 | 214 (99.5) | 1 (0.5) | 0 | 0 |
| Self-neglect | 242 (99.6) | 0 | 1 (0.4) | 0 | 213 (99.1) | 1 (0.5) | 1 (0.5) | 0 |
| Grandiosity | 243 (100) | 0 | 0 | 0 | 214 (99.5) | 1 (0.5) | 0 | 0 |
| Hostility | 243 (100) | 0 | 0 | 0 | 214 (99.5) | 1 (0.5) | 0 | 0 |

**KEY:**

None= No neuropsychiatric symptoms; Mild = Mild neuropsychiatric symptoms; Mod = Moderate neuropsychiatric symptoms; Sev = severe neuropsychiatric symptoms
