# Supplementary figures and images for: Neuropsychiatric manifestations among HIV-1 infected African patients receiving efavirenz-based cART with or without tuberculosis treatment containing rifampicin
Source: Eur J Clin Pharmacol. 2018 Jul 12;74(11):1405–15. doi: 10.1007/s00228-018-2499-0 (PMC6182598; doi:10.1007/s00228-018-2499-0)

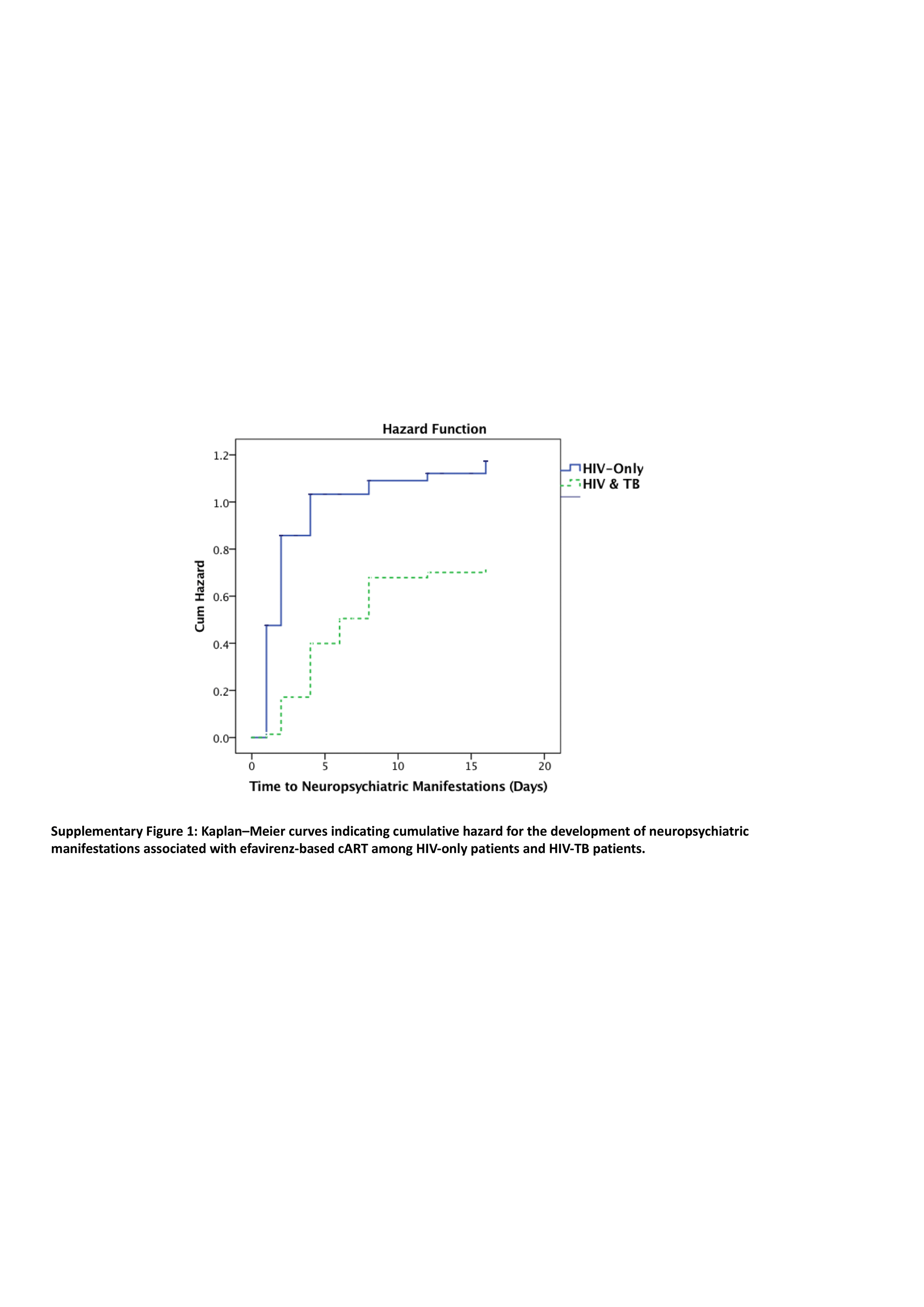

Supplement: Supplementary file 2 — (PNG 278 kb) [file 228_2018_2499_Fig2_ESM.png]
